# Significant association between Let-7-KRAS rs712 G > T polymorphism and cancer risk in the Chinese population: a meta-analysis

**DOI:** 10.18632/oncotarget.14672

**Published:** 2017-01-16

**Authors:** Xin-Ya Du, Yuan-Yuan Hu, Chun Xie, Chun-Yan Deng, Cai-Yun Liu, Zhi-Guo Luo, Yu-Ming Niu, Ming Shen

**Affiliations:** ^1^ Department of Stomatology, People's Hospital of New District Longhua Shenzhen, Shenzhen 518109, China; ^2^ Department of Stomatology, Taihe Hospital, Hubei University of Medicine, Shiyan 442000, China; ^3^ Intensive Care Unit, Taihe Hospital, Hubei University of Medicine, Shiyan 442000, China; ^4^ Center for Evidence-Based Medicine and Clinical Research, Taihe Hospital, Hubei University of Medicine, Shiyan 442000, China; ^5^ Department of Clinical Oncology, Taihe Hospital, Hubei University of Medicine, Shiyan 442000, China; ^6^ Jiangsu Key Laboratory of Oral Diseases, Nanjing Medical University, China; ^7^ Department of Dental Implant, Affiliated Hospital of Stomatology, Nanjing Medical University, Nanjing 210029, China

**Keywords:** let-7, KRAS, polymorphism, cancer

## Abstract

Association between let-7-KRAS rs712 polymorphism and cancer risk was inconsistent. We therefore conducted this meta-analysis to clarify the association between let-7-KRAS rs712 polymorphism and cancer risk with STATA 14.0 software. A systemic literature search in online databases (PubMed, Embase, CNKI and Wanfang database) was preformed to obtain relevant articles. A total of 13 case-control studies involving 3,453 patients and 4,470 controls were identified up to May 16, 2015. The pooled results indicated that significantly increased risk were observed in Chinese population in T vs. G (OR = 1.21, 95% CI = 1.03–1.42) and TT vs. GG + GT genetic models (OR = 1.69, 95% CI = 1.17–2.42). Sensitivity analysis was conducted and the result without heterogeneity showed significant associations in all five genetic models. Subgroup analyses of cancer type indicated a similar result in digestive cancer (for T vs. G: OR = 1.41, 95% CI = 1.26–1.57; GT vs. GG: OR = 1.24, 95% CI = 1.07–1.43; TT vs. GG: OR = 2.53, 95% CI = 1.86–3.44; GT + TT vs. GG: OR = 1.36, 95% CI = 1.19–1.56; TT vs. GG + GT: OR = 2.35, 95% CI = 1.73–3.19). In summary, these evidences demonstrate that let-7-KRAS rs712 G > T polymorphism might be associated with digestive system cancer risk in the Chinese population.

## INTRODUCTION

Cancer, one of the major common malignant diseases contributes to death worldwide, which has become an important healthy problem [1]. In China, accompanied with the accelerated deterioration of the environment and the population aging, the incidence of cancer has been rising in recent ten year. Today, cancer has become the leading cause of death in China, more than 4292,000 new cancer patents and 2814,000 deaths would occur in 2015 [2]. Dysfunction, deformity, and mental stress have seriously reduced the quality of life of cancer patients. Furthermore, the increasing medical costs have become a heavy economic burden on families and society [3, 4]. Unfortunately, the development mechanism of cancer has not been clearly explained, although numerous epidemiological and molecular biology researches has shown that live habits, nutritional intake, mental state, and chronic inflammation are contributed to cancer risk [5].

To date, a large numbers of studies have indicated that genetic abnormity maybe result in tumorigenesis [6, 7]. MicroRNA always consist of short, single-stranded, noncoding RNAs with 20–22 nucleotides long, which could take part in the genetic post-transcriptional regulation and influenced the cell procedures of differentiation, proliferation, apoptosis [8]. Lethal-7 (let-7) is the earliest discovered microRNA family, which is an important genetic regulators through controlling cancer oncogene expression by binding to the complementary elements in the 3′ untranslated regions (UTRs) of their target messenger RNAs (mRNAs) [9]. Let-7 could decrease KRAS expression through a let-7-KRAS binding located at specific sites of the 3′ UTRs of KRAS, which has been proved one of the most frequently activated oncogenes [10].

Gene mutation, including single-nucleotide polymorphisms (SNPs), such as interleukin gene family polymorphisms and microRNA polymorphisms has been proved to be associated with cancer risk. Regarding KARS gene, several common SNPs located at the 3′-UTR region have been identified, such as rs712 G > T polymorphism. A recent study with luciferase vector reporter system demonstrated that the let-7 would decrease the activity of KRAS, but the rs712 minor allele would compromise the interaction between let-7g and KRAS 3′-UTR [11].

From 2014, two meta-analyses were conducted, only 6 case-control studies were included in both meta-analyses [12, 13]. Today, more than ten studies that assessed the association between rs712 G > T polymorphism and cancer risk published. Until now, most of the studies focused on Chinese population without consistent conclusion. Therefore, we performed this updated meta-analysis to further investigate an accurate association between rs712 G > T polymorphism and cancer risk in the Chinese population.

## RESULTS

### Study characteristics

**Figure 1:**
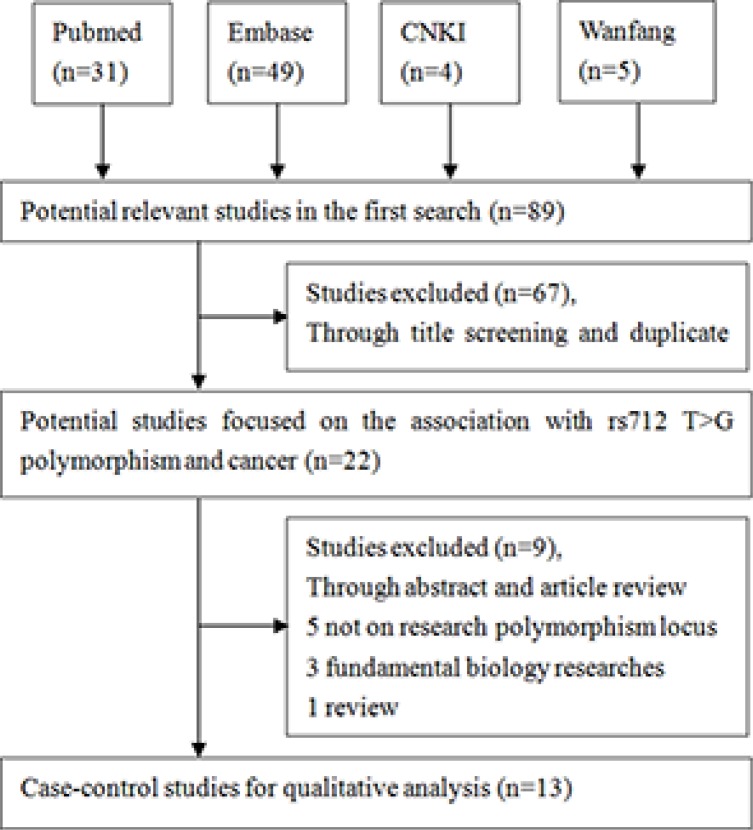
Flow diagram of the study selection process

A total of 89 studies were identified initially. Figure 1 showed the selecting procession of studies step by step. After reviewed the titles and abstracts, 67 articles were excluded. Through reading full texts, we deleted another 9 articles. Finally, 13 articles involving 3,453 patients and 4,470 controls were selected in our meta-analysis based on the inclusion criteria [11, 14–25]. Among them, 5 articles on digestive system cancer with 1,798 patients and 2,145 controls [15, 17, 22–24], which included three studies on Colorectal cancers, one study on gastric cancer and the other study on Hepatocellular cancer. 3 articles on head and neck cancer with 593 patients and 850 controls [16, 18, 19], 2 articles on lung cancer with 215 patients and 502 controls [11, 14], 2 articles on cervical cancer with 619 patients and 722 controls [20, 21, 25], one article breast cancer with 228 patients and 251 controls [25]. In term of genotyping method, 11 studies used polymerase chain reaction-restriction fragment length polymorphism (PCR-RFLP), one study adopted Real-time PCR [11] and another study used iMLDR [22] method. The genotype distributions in controls are all satisfy with HWE. All included characteristics of each study were summarized in Table 1.

**Table 1:** Characteristics of case-control studies on Let-7-KRAS rs712 G > T polymorphism and cancer risk included in the meta-analysis

First author	Year	Genotype method	Control design	Case	Control	Genotype distribution							P for HWE	MAF		Location	NOS
						Case				Control							
						GG	GT	TT		GG	GT	TT		Case	Control		
Peng	2010	PCR-RFLP	HB	83	80	49	31	3		51	25	4	0.68	0.22	0.21	Lung	7
Li	2013	PCR-RFLP	HB	181	674	105	60	16		442	211	21	0.49	0.25	0.19	Gastric	8
Yan	2013	PCR-RFLP	HB	153	204	83	56	14		137	61	6	0.80	0.27	0.18	Glioma	6
Pan1	2014	PCR-RFLP	HB	339	313	188	125	26		203	100	10	0.58	0.26	0.19	Colorectal	8
Pan2	2014	PCR-RFLP	HB	188	356	112	64	12		201	138	17	0.34	0.23	0.24	Nasopharyngeal	7
Jin	2014	PCR-RFLP	HB	252	290	154	84	14		183	92	15	0.44	0.22	0.21	Thyroid	7
Ni	2015	PCR-RFLP	HB	204	218	112	73	19		145	67	6	0.60	0.27	0.18	Cervical	6
Liang	2015	PCR-RFLP	HB	415	504	257	144	14		327	163	14	0.23	0.21	0.19	Cervical	8
Hu	2015	Real-time PCR	HB	132	422	22	38	72		12	132	278	0.43	0.69	0.82	Lung	7
Dai	2015	iMLDR	HB	430	430	253	145	32		283	130	17	0.67	0.24	0.19	Colorectal	8
Xiong	2015	PCR-RFLP	PB	262	252	150	92	20		162	79	11	0.73	0.25	0.20	Hepatocellular	9
Jiang	2015	PCR-RFLP	HB	586	476	372	176	38		331	133	12	0.75	0.22	0.16	Colorectal	8
Huang	2015	PCR-RFLP	HB	228	251	155	65	8		173	71	7	0.93	0.18	0.17	Breast	7

^a^HWE in control

Abbreviation: MAF: Minor allele frequency in control group.

HB: Hospital-based; PB: Population-based.

### Quantitative analysis

**Figure 2:**
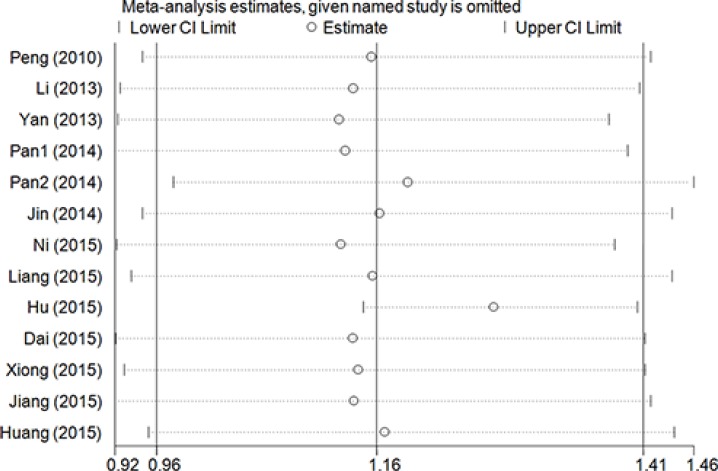
Sensitivity analysis through deleting each study to reflect the influence of the individual dataset to the pooled ORs in GT+TT vs. GG model of rs712 G > T polymorphism

**Figure 3:**
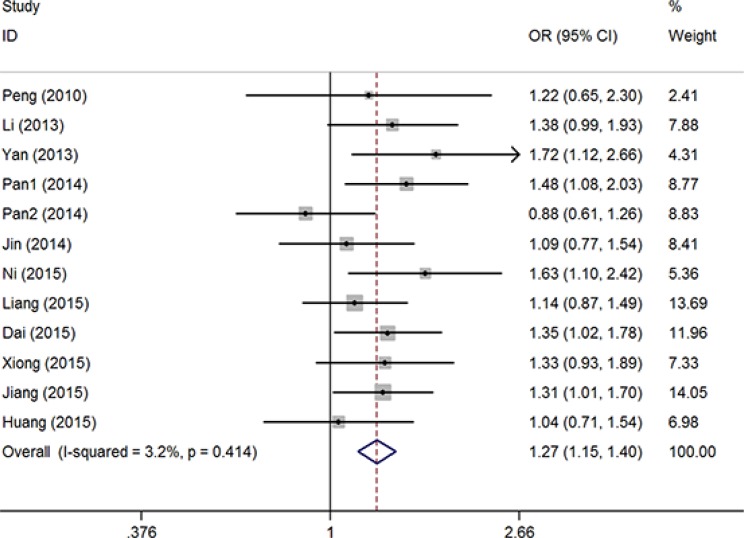
OR and 95% CIs of the associations between rs712 G > T polymorphism and cancer risk in GT + TT vs. GG model

**Figure 4:**
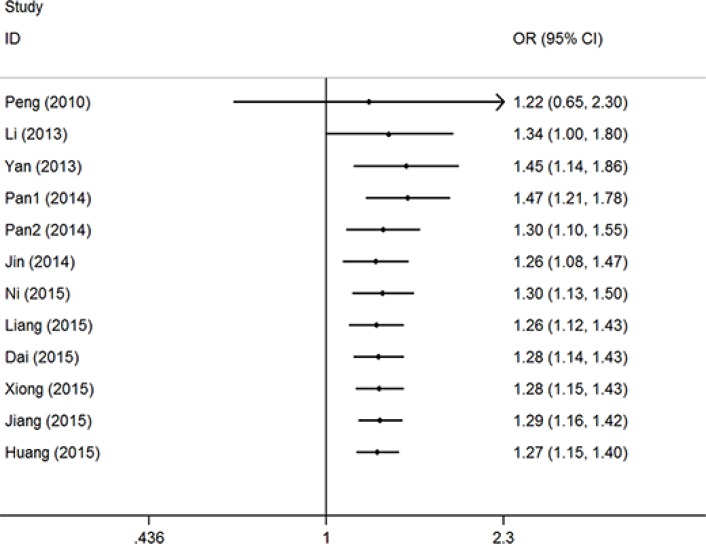
Cumulative meta-analyses according to publication year in GT + TT vs. GG model of rs712 G > T polymorphism

Overall, significantly elevated risks were observed with combined studies in allele contrast model (T vs. G: OR = 1.21, 95% CI = 1.03–1.42, *P* = 0.03, I^2^ = 75.8%) and recessive model (TT vs. GG + GT: OR = 1.69, 95% CI = 1.17–2.42, *P* = 0.01, I^2^ = 67.4%). Obviously heterogeneities were found in all analyzed genetic models. Sensitive analysis were conducted though deleting each study one by one, and the results indicated that the report by Hu et al. [11] maybe the critical important factor which result in these heterogeneities (Figure 2). Then, significant associations presented in all five genetic models with obviously reduced heterogeneities without the study of Hu et al. [11] (T vs. G: OR = 1.30, 95% CI = 1.20–1.41, *P* < 0.01, I^2^ = 33.7%; GT vs. GG: OR = 1.18, 95% CI = 1.07–1.31, *P* < 0.01, I^2^ = 0%; TT vs. GG: OR = 2.07, 95% CI = 1.65–2.59, *P* < 0.01, I^2^ = 24.8%; GT + TT vs. GG: OR = 1.27, 95% CI = 1.15–1.41, *P* < 0.01, I^2^ = 3.2%, (Figure 3); TT vs. GG + GT: OR = 1.96, 95% CI = 1.57–2.44, *P* < 0.01, I^2^ = 14.8%) (Supplementary Figure 1). Accumulative analysis indicated that the increased risk with rs712 T > G polymorphism could be found in 2013, and the result was further confirmed with added researches (Figure 4 for GT + TT vs. GG model) (Supplementary Figure 2).

Subgroup analysis based on cancer location, control resource, and genotype methods were conducted. Significant cancer risk were also found without heterogeneity in the subgroup of digestive system cancer (T vs. G: OR = 1.41, 95% CI = 1.26–1.57, *P* < 0.01, I^2^ = 0%; GT vs. GG: OR = 1.24, 95% CI = 1.07–1.43, *P* < 0.01, I^2^ = 0%; TT vs. GG: OR = 2.53, 95% CI = 1.86–3.44, *P* < 0.01, I^2^ = 0%; GT + TT vs. GG: OR = 1.36, 95% CI = 1.19–1.56, *P* < 0.01, I^2^ = 0%; TT vs. GG + GT: OR = 2.35, 95% CI = 1.73–3.19, *P* < 0.01, I^2^ = 0%). Furthermore, other significant increased associations were also found in the subgroup analysis by control resource, and genotype methods (Table 2).

**Table 2:** Summary ORs and 95% CI of Let-7-KRAS rs712 G > T polymorphisms and cancer risk

	N*	T vs. G					GT vs. GG					TT vs. GG					GT+TT vs. GG					TT vs. GG+GT			
Unadjusted		OR	95% CI	P	I2(%)		OR	95% CI	P	I2(%)		OR	95% CI	P	I2(%)		OR	95% CI	P	I2(%)		OR	95% CI	P	I2(%)
Total	13	1.21	1.03-1.42	0.03	75.8		1.11	0.93-1.31	0.24	62.0		1.61	0.98-2.65	0.06	79.8		1.16	0.96-1.41	0.12	72.8		1.69	1.17-2.42	0.01	67.4
Sensitive analysis^#^	12	1.30	1.20-1.41	<0.01	33.7		1.18	1.07-1.31	<0.01	0		2.07	1.65-2.59	<0.01	24.8		1.27	1.15-1.41	<0.01	3.2		1.96	1.57-2.44	<0.01	14.8
Location																									
Digestive system	5	1.41	1.26-1.57	<0.01	0		1.24	1.07-1.43	<0.01	0		2.53	1.86-3.44	<0.01	0		1.36	1.19-1.56	<0.01	0		2.35	1.73-3.19	<0.01	0
Head and neck	3	1.20	0.86-1.67	0.29	70.6		1.09	0.79-1.50	0.61	49.8		1.65	0.82-3.32	0.16	52.7		1.16	0.81-1.67	0.42	64.2		1.58	0.86-2.92	0.14	40.1
Lung	2	0.72	0.34-1.56	0.41	84.1		0.46	0.06-3.59	0.46	93.		0.29	0.06-1.52	0.14	73.9		0.43	0.05-3.41	0.42	94.6		0.63	0.43-0.92	0.02	0
Cervical	2	1.35	0.90-2.02	0.14	75.5		1.21	0.96-1.52	0.11	0		2.20	0.70-6.94	0.18	72.0		1.32	0.93-1.88	0.12	54.8		2.03	0.70-5.90	0.19	68.4
Breast	1	1.06	0.76-1.48	0.73	NA		1.02	0.68-1.52	0.92	NA		1.28	0.45-3.60	0.65	NA		1.04	0.71-1.54	0.83	NA		1.27	0.45-3.55	0.65	NA
Design																									
HB	12	1.20	1.00-1.43	0.05	77.6		1.09	0.91-1.31	0.34	65.0		1.58	0.92-2.72	0.10	81.4		1.15	0.93-1.41	0.20	74.9		1.68	1.14-2.49	0.01	69.9
PB	1	1.33	0.99-1.78	0.06	NA		1.24	0.85-1.80	0.26	NA		1.94	0.90-4.18	0.09	NA		1.33	0.93-1.89	0.12	NA		1.80	0.84-3.83	0.13	NA
Genotype method																									
PCR-RFLP	11	1.29	1.18-1.41	<0.01	39.0		1.17	1.05-1.31	<0.01	0		2.06	1.62-2.63	<0.01	31.6		1.26	1.14-1.40	<0.01	10.5		1.96	1.54-2.49	<0.01	22.5
Others	2	0.83	0.31-2.21-	0.71	96.0		0.46	0.06-3.49	0.45	95.7		0.55	0.04-7.79	0.66	96.7		0.46	0.05-4.01	0.48	96.7		1.08	0.35-3.31	0.90	89.6
**Adjusted**																									
Total	12	1.32	1.18-1.79	<0.01	45.6		1.20	1.08-1.34	<0.01	0		1.58	0.93-2.70	0.09	82.0		1.12	0.73-1.70	0.61	87.0		1.94	1.26-3.00	<0.01	16.6
Sensitive analysis^#^	10	1.32	1.18-1.79	<0.01	45.6		1.20	1.08-1.34	<0.01	0		1.99	1.49-2.66	<0.01	32.4		1.39	1.21-1.61	<0.01	21.8		1.94	1.26-3.00	<0.01	16.6
Location																									
Digestive system	5	1.41	1.27-1.55	<0.01	0		1.30	1.12-1.51	<0.01	0		2.42	1.76-3.33	<0.01	0		1.44	1.16-1.79	<0.01	14.0		2.45	1.44-4.15	<0.01	0
Head and neck	3	1.20	0.86-1.67	0.29	71.2		1.09	0.79-1.51	0.60	50.4		1.65	0.82-3.33	0.16	52.7		1.73	1.12-2.67	0.01	NA		NA	NA	NA	NA
Lung	1	NA	NA	NA	NA		NA	NA	NA	NA		0.14	0.07-0.29	<0.01	NA		0.15	0.07-0.32	<0.01	NA		NA	NA	NA	NA
Cervical	2	1.69	1.22-2.34	<0.01	NA		1.20	0.96-1.51	0.12	0		2.19	0.69-6.96	0.18	72.3		1.32	0.92-1.88	0.13	55.6		1.22	0.57-2.60	<0.01	NA
Breast	1	0.94	0.65-1.35	0.74	NA		0.98	0.66-1.46	0.92	NA		0.78	0.28-2.19	0.64	NA		NA	NA	NA	NA		NA	NA	NA	NA
Design																									
HB	11	1.32	1.16-1.50	<0.01	51.6		1.18	1.05-1.31	<0.01	0		1.52	0.86-2.69	0.15	83.3		1.01	0.62-1.65	0.97	88.9		1.85	0.85-4.01	0.12	57.7
PB	1	1.35	1.02-1.79	0.04	NA		1.64	1.08-2.50	0.02	NA		12.56	1.05-6.25	0.04	NA		1.75	1.16-2.65	<0.01	NA		2.08	0.87-4.96	0.10	NA
Genotype method																									
PCR-RFLP	10	1.31	1.14-1.50	<0.01	49.3		1.19	1.06-1.33	<0.01	3.7		2.00	1.55-2.59	<0.01	39.1		1.39	1.21-1.61	<0.01	21.8		1.94	1.26-3.00	<0.01	16.6
Others	2	1.42	1.21-1.66	<0.01	NA		1.31	0.93-1.84	0.12	NA		0.52	0.04-6.70	0.62	96.6		0.15	0.07-0.32	<0.01	NA		NA	NA	NA	NA

^*^ Numbers of comparisons

^a^ Test for heterogeneity

^#^ Sensitive analysis without the report of Hu et al.

**Figure 5:**
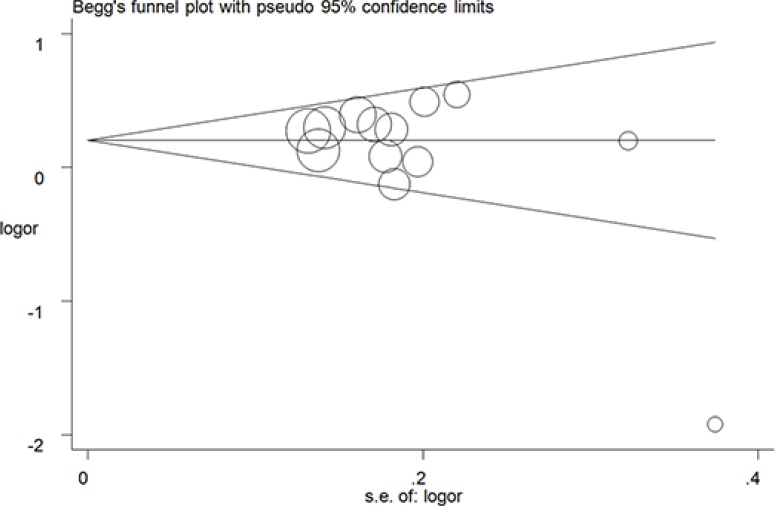
Funnel plot analysis to detect publication bias for GT + TT vs. GG model of rs712 G > T polymorphism. Circles represent the weight of the studies

Begg's tests were performed with funnel plot to assess publication bias. No apparently asymmetry was found (Figure 5 for GT + TT vs. GG model) (Supplementary Figure 3), and there results were further guaranteed by Egger's test (T vs. G, *P* = 0.62; GT vs. GG: *P* = 0.08; TT vs. GG, *P* = 0.82; GT + TT vs. GG, *P* = 0.08; TT vs. GG + GT, *P* = 0.08).

## DISCUSSION

In 2012, there were more than 14.1 million new cancer patient and 8.2 million deaths worldwide [26]. Today, cancer is still the most common malignant disease due to death and disability. The incidence of cancer in the developing countries is gradually increasing, with the aging of the population and the deterioration of environmental factors. China has the largest population in the world, and the incidence of cancer in China has been high, leading to a decline in quality of the living standards [2]. The occurrence of cancer is the result of interaction of various factors. Diet, living habits, cell abnormalities, gene mutations are one of the factors that lead to the development of tumor. Different regions, racial diversity, may be the cause of the changes in cancer susceptibility.

Let-7 family has several members, recent studies have found that the Let-7 could function as a tumor suppressor during the development of solid tumors through inhibiting cancer proliferation by targeting some oncogenes/antioncogenes, including KRAS gene Chun-Yan Deng [27, 28]. KRAS is one of the critical oncogene, which locates at 12p12.1. Some single nucleotide polymorphisms (SNPs) residing in 3′ UTRs of KRAS gene have been found effected cancer risk through altering the activation of KRAS gene. Such as the KRAS-LCS (rs61764370) polymorphism had been proved to influence the KRAS transcription, resulting in an increased KRAS expression in non-small cell lung cancer [29]. Today, the interaction mechanism of many polymorphism locus located in different genes sites is more and more attracting our attentions. A large number studies had suggested that, many gene mutations, such as BRAF, E-Cadherin, and TP53 genes play an important role during the process of cancer development. The synergistic effects of these polymorphisms maybe initiate the procedure of abnormal changes of normal cells, and to accelerate the formation of the tumor solid [30–32].

Recently, the SNP of rs712 G > T polymorphism in the let-7-KRAS binding site has been reported and drawn more attentions. Kim et al. reported that the rs712 G allele would downgrade more than 15% activity compared with rs712 T allele with luciferase reporter *in vitro* [33]. In 2010, Peng et al. conducted the first case-control study and didn't found any significant association between the patients with lung cancer and healthy controls in a Chinese population [14]. Since then, a lot of case-control studies have been conducted, but the conclusions were not consistent. In 2014, Ying et al. conducted the first meta-analysis including only six case-controls studies. The results demonstrated that no significant association was found between rs712 polymorphism and cancer susceptibility in the overall population, but the subgroup analysis found that the allele T (T vs. G: OR = 1.33, 95% CI = 1.08–1.64, *P* = 0.01) and dominant genotype (GT + TT vs. GG: OR = 1.30, 95% CI = 1.11–1.55, *P* < 0.01) would increase the risk of cancer in Chinese population. Moreover, Zhao et al conducted another meta-analysis and obtained the similar results with the same six studied. However, there were only five case-controls of Chinese population were included in the two meta-analysis. No further subgroup analysis of cancer location, control resource, and genotype methods was conducted owe to the limited number of researches and small sample size. To our knowledge, meta-analysis is a scientific statistical method to draw more precise results through expanding sample with as much as possible homogeneity studies. Today, there are seven new researcher articles on the association between the rs712 G > T polymorphism and cancer risk had been published, and all these seven articles focused on Chinese population. So, we conducted the updated systematic meta-analysis to answer this question “Does this mutation of rs712 G > T increase the susceptibility of cancer in Today's China population?” fatherly. Our results demonstrate that the rs712 G > T polymorphism might be increase the digestive system cancer risk in the Chinese population. Furthermore, there were three studies focused on colorectal cancer and the results of meta-analysis indicated that the rs712 G > T might be associate with increased colorectal cancer risk (T vs. G: OR = 1.41, 95% CI = 1.23–1.61, *P* < 0.01, I^2^ = 0%; GT vs. GG: OR = 1.25, 95% CI = 1.05–1.48, *P* = 0.01, I^2^ = 0%; TT vs. GG: OR = 2.52, 95% CI = 1.71–3.71, *P* < 0.01, I^2^ = 0%; GT + TT vs. GG: OR = 1.37, 95% CI = 1.16–1.61, *P* < 0.01, I^2^ = 0%; TT vs. GG + GT: OR = 2.34, 95% CI = 1.59–3.42, *P* < 0.01, I^2^ = 0%). More researches, larger sample size and further subgroup analysis were included in our meta-analysis, in order to enhance statistical efficiency and increase the accuracy of the effect. Furthermore, we extracted the adjusted data (OR and 95% CI) and pooled them for investigating the interactions between genetic polymorphisms and environmental risk factors. These results were almost consistent with the former results that we conducted with unadjusted data.

In this meta-analysis, our result demonstrated that the G allele would increase the cancer susceptibility in Chinese population with some apparently heterogeneities. Through the sensitivity analysis, we found the data of Hu et al. maybe the main reason leading to heterogeneity through reviewing of the minor allele frequency of controls [11]. The results indicated that the frequency distribution of minor allele frequency in his article was higher than other studies. And the heterogeneity was alleviated through deleting the data of Hu et al. In the next subgroup analysis, the pooled results indicated that the rs712 G > T polymorphism was associated with the development of digestive system cancer. This difference suggested that gene mutations may be associated with a specific susceptibility to some cancer, which may provide us an important approach to early screening and prevention in the future.

Moreover, some limitations of this meta-analysis should to be raised. Firstly, only one SNP locus (rs712 G > T) was analysis in this article, and the statistical calculations were conducted without other risk factors, such as lifestyle, bad habits (smoking and drinking), environmental deterioration, and other gene mutations. Secondly, the racial bias could not be eliminated due to only Chinese population was included in this research. And the conclusion should be tested before applying to other populations again. Third, although we have included thirteen studies, the sample size is still insufficient, which could made some deviations from the truly results.

In conclusion, our meta-analysis suggest that the rs712 G > T polymorphism is associated with an increased cancer risk in Chinese population, especially in digestive system cancer. Additional studies with large sample sizes in other ethnic populations are needed to guarantee our findings further.

## MATERIALS AND METHODS

This meta-analysis was conducted following Preferred Reporting Items for Systematic Reviews and Meta-Analyses (PRISMA) statement [34]. All included data were collected from published studies, and no ethical issues were involved.

### Search strategy

Four electronic databases (PubMed, Embase, CNKI and Wanfang database) were searched with following terms: “let”, “KRAS”, “polymorphism”, and “cancer” through the review of “let-7[All Fields] AND (“proto-oncogene proteins p21(ras)”[MeSH Terms] OR (“proto-oncogene”[All Fields] AND “proteins” [All Fields] AND “p21(ras)”[All Fields]) OR “proto-oncogene proteins p21(ras)”[All Fields] OR “kras”[All Fields]) AND (“polymorphism, genetic”[MeSH Terms] OR (“polymorphism”[All Fields] AND “genetic”[All Fields]) OR “genetic polymorphism”[All Fields] OR “polymorphism”[All Fields]) AND (“neoplasms”[MeSH Terms] OR “neoplasms”[All Fields] OR “cancer”[All Fields])”, up to 10 June, 2016. Only studies written with English and Chinese were selected.

### Study selection

All included studies according to these inclusion criteria: (1) case-control studies; (2) research focus on rs712 G > T polymorphism and cancer risk; and (3) the publication with adequate information to calculate the odds ratio (OR) and 95% confidence interval (CI). The exclusion criteria: (1) review studies; (2) molecular fundamental studies; (3) data not about the related locus or with in sufficient outcome; and (4) duplicated or overlapping data of the same author or issue.

### Data extraction

Two independent reviewers (Du and Xie) review and extracted the relevant the data from all included studies: the first author's name, study published date, sources of controls, genotyping method, cancer location, adjusted OR and its 95% CI, MAF (Minor allele frequency) in cases and controls, and the distributed number of genotypes in cases and controls. Quality assessment of included studies was evaluated with modified Newcastle-Ottawa scale (NOS) by two authors, and the scores ranged from 0 points (worst) to 9 points (best) (Table 3) [35].

**Table 3:** Scale for quality assessment

Criteria	Score
**Representativeness of cases**	
Consecutive/randomly selected form case population with clearly defined sampling frame	2
Consecutive/randomly selected form case population without clearly defined sampling frame or with extensive	1
Not described	0
**Source of controls**	
Population- or Healthy-based	2
Hospital-bases	1
Not described	0
**Hardy-Weinberg equilibrium in controls**	
Hardy-Weinberg equilibrium	2
Hardy-Weinberg disequilibrium	1
**Genotyping examination**	
Genotyping done under “blinded” condition	1
Unblinded done or not mentioned	0
**Association assessment**	
Assess association between genotypes and cancer with appropriate statistics and adjustment for confounders	2
Assess association between genotypes and cancer with appropriate statistics and without adjustment for confounders	1
Inappropriate statistics used	0

### Statistical analysis

First, Hardy-Weinberg equilibrium (HWE) in controls of every included study was calculated by Chi-square test. Second, Crude ORs with 95% CIs were used to assess the association between rs712 G > T polymorphism and cancer risk. Five genetic models were analyses, involving allele contrast (T vs. G), co-dominant (GT vs. GG and TT vs. GG), dominant (GT + TT vs. GG) and recessive (TT vs. GG + GT) models. Stratified assessments were calculated based on cancer location, control resource, and genotype methods. Heterogeneity between studies was calculated with the *I*^2^ value and Cochran's *Q* test. The fixed-effect model (the Mantel-Haenszel method) was applied when the I2 value less than 50% and *P* > 0.10 for the *Q* test; otherwise, a random effects model (the DerSimonian and Laird method) was adopted. Third, cumulative meta-analyses were conducted to identify a possible trend of the pooled results with new studies added. Sensitivity analyses were also conducted to examine the stability of the results through deleting each study one by one. Finally, publication bias was assessed with Begg's funnel plot and Egger's test. All statistical analyses were performed using STATA version 14.0 (Stata Corporation, College Station, TX, USA). A *P value* < 0.05 was considered statistically significant.

## SUPPLEMENTARY MATERIALS FIGURES


