# Supplementary material for: Significant association between Let-7-KRAS rs712 G > T polymorphism and cancer risk in the Chinese population: a meta-analysis
Source: Oncotarget. 2017 Jan 16;8(8):13863–71. doi: 10.18632/oncotarget.14672 (PMC5355145; doi:10.18632/oncotarget.14672)
Supplement: Supplementary file 1 [file oncotarget-08-13863-s001.pdf]

Significant association between Let-7-KRAS rs712 G > T polymorphism and cancer risk in the Chinese population: a meta-analysis

Supplementary Materials

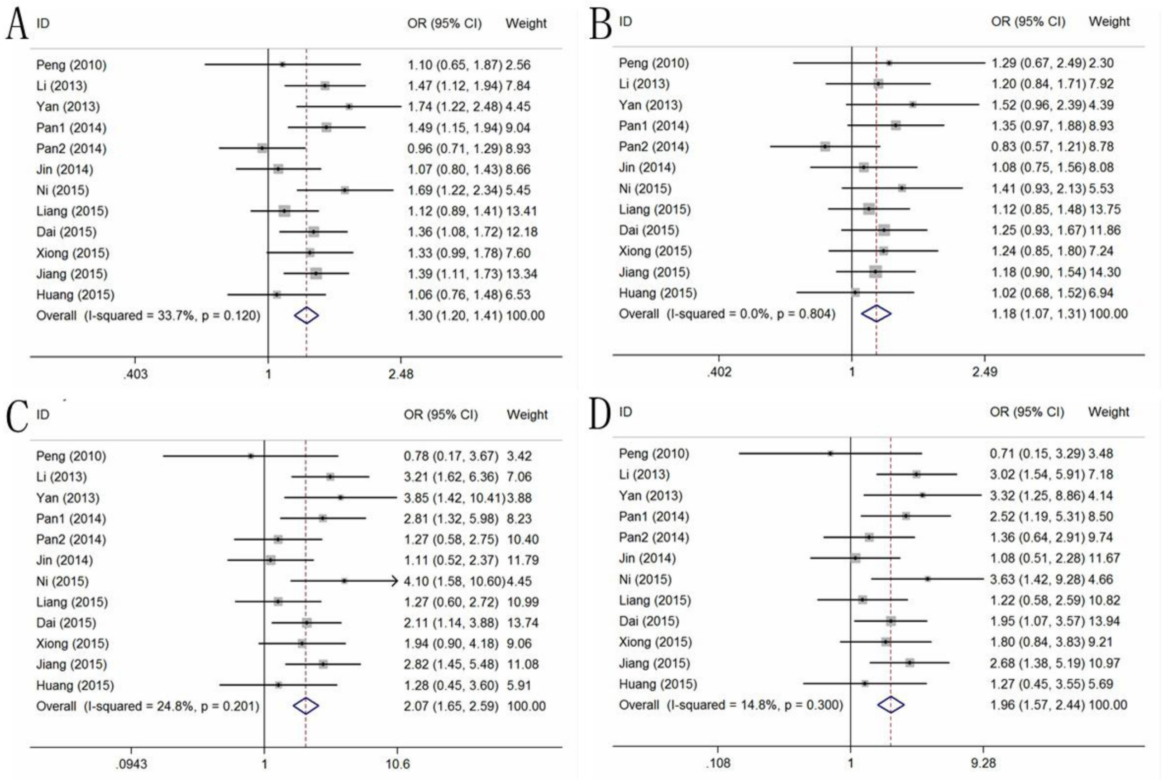

Supplementary Figure 1: OR and 95% CIs of the associations between rs712 G > T polymorphism and cancer risk ((**A**) for T vs. G model; (**B**) for GT vs. GG model; (**C**) for TT vs. GG model; (**D**) for TT vs. GG + GT model).

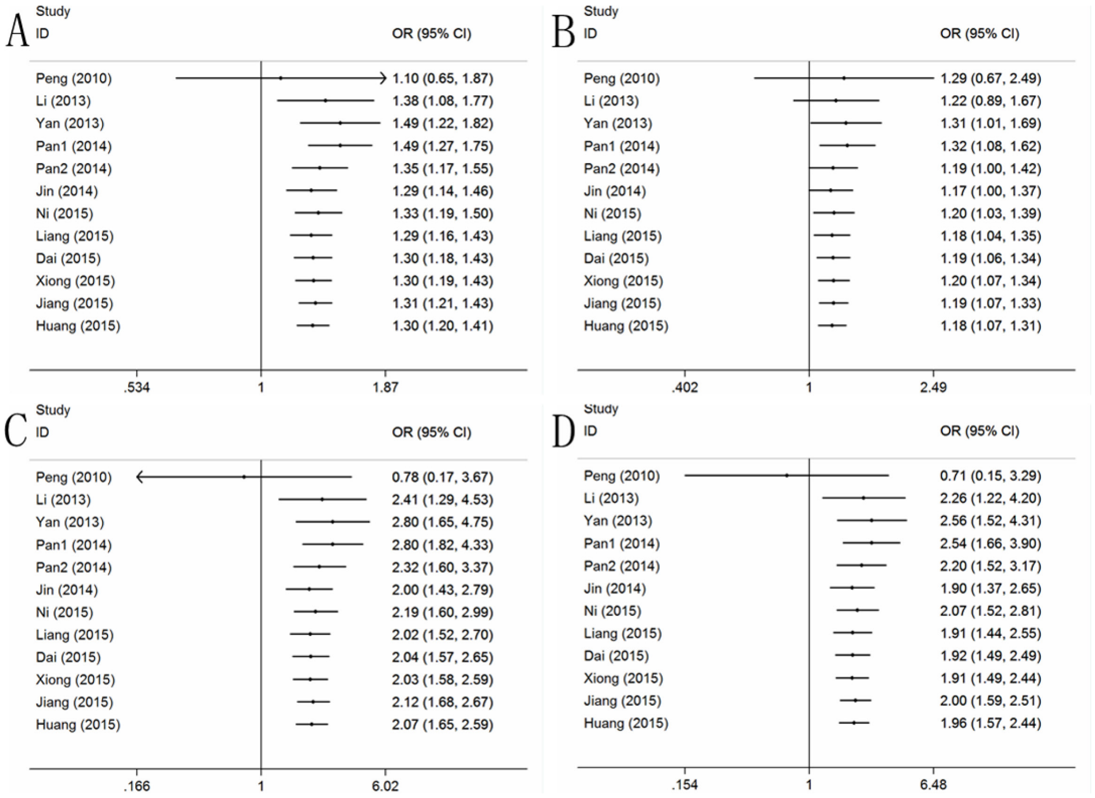

Supplementary Figure 2: Cumulative meta-analyses according to publication year in rs712 G > T polymorphism ((**A**) for T vs. G model; (**B**) for GT vs. GG model; (**C**) for TT vs. GG model; (**D**) for TT vs. GG + GT model).

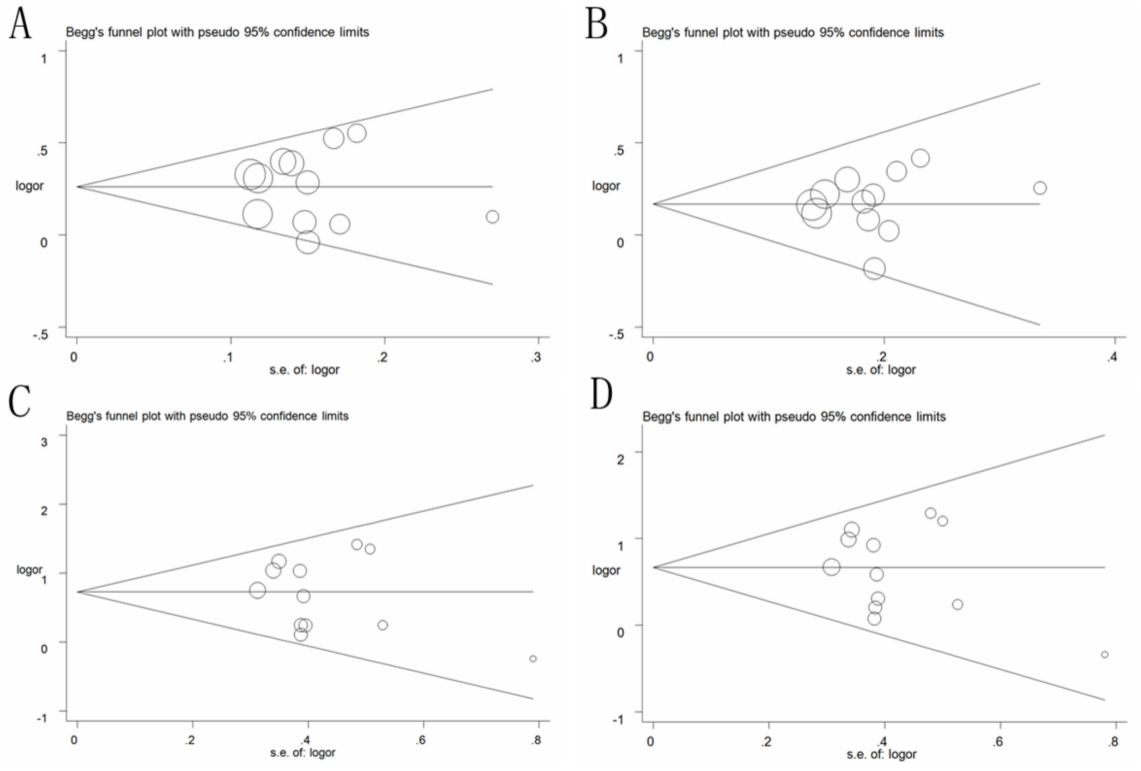

Supplementary Figure 3: Funnel plot analysis to detect publication bias in rs712 G > T polymorphism ((**A**) for T vs. G model; (**B**) for GT vs. GG model; (**C**) for TT vs. GG model; (**D**) for TT vs. GG + GT model). Circles represent the weight of the studies.
